# Prevalence of unintended pregnancy and associated factors among pregnant women with disabilities in Ethiopia: from the social model of disability perspective

**DOI:** 10.3389/fgwh.2024.1458664

**Published:** 2024-12-16

**Authors:** Abebe Alemu Anshebo, Yilma Markos Larebo, Sujit Behera, Natarajan Gopalan

**Affiliations:** ^1^ Department of Epidemiology and Public Health, School of Life Science, Central University of Tamil Nadu, Thiruvarur, India; ^2^ Department of Midwifery, College of Medicine and Health Science, Wachemo University, Hosanna, Ethiopia; ^3^ Department of Public Health, College of Medicine and Health, Wachemo University, Hosanna, Ethiopia

**Keywords:** unintended, pregnancy, women, disabilities, Ethiopia

## Abstract

**Background:**

In low-income countries, women with disabilities face numerous challenges in accessing sexual and reproductive health services and experience high unintended pregnancy rates and adverse pregnancy outcomes, with 42% of cases ending in abortion. However, little is known about unintended pregnancy among women with disabilities in Ethiopia. Therefore, this study aimed to assess the prevalence of unintended pregnancy and associated factors among women with disabilities in the Central Regional State of Ethiopia.

**Methods:**

A community-based cross-sectional study was conducted from December 2023 to February 2024, and multistage random sampling was used to enroll 572 study participants. The sample size was proportionally allocated to each zone, district and kebele. The Kobo Toolbox was used for data collection and cleaning, and the Statistical Package for Social Science version 26 was used for analysis. The multivariable analysis was used to identify the factors significantly associated with unintended pregnancy using an adjusted odds ratio (AOR), a 95% confidence interval (CI), and a *p*-value less than 0.05.

**Results:**

The prevalence of unintended pregnancy was 43.8% (95% CI: 39.5, 47.8) in the Central Ethiopia Regional State, Ethiopia. The significantly associated factors were household size (AOR = 4.6, 95% CI: 2.6, 7.9), awareness of pregnancy intention (AOR = 2.4, 95% CI:1.4, 4.1), domestic violence (AOR = 5.9, 95% CI: 3.4, 10.4), accessibility of service (AOR = 2.4, 95% CI: 1.2, 5.4), discrimination by care providers (AOR = 2.1, 95% CI: 1.5, 2.9), disability-unfriendly health facility structure (AOR = 1.5, 95% CI: 1.2, 2.40), and negative community attitudes (AOR = 2.7, 95% CI: 1.7, 4.3). The overall direction of the associated variables’ effect was positive.

**Conclusion:**

This study sheds light on the need for disability-inclusive and sensitive healthcare services. Therefore, to achieve universal access to sexual and reproductive healthcare targeted under the Sustainable Development Goals, the Minister of Women and Social Affairs, Ministry of Health, Regional Health Bureau, and health facilities authorities should pay attention to improving awareness on pregnancy intention and domestic violence and ensuring accessible, inclusive, and equitable maternal health care for women with disabilities.

## Introduction

An unintended pregnancy is one that occurs for a woman who does not plan to have any or more children, or that was mistimed; it happened earlier than desired (1, 2). Almost half of all pregnancies worldwide are unintended, totalling 121 million yearly, or 331,000 per day (2). In Sub-Saharan Africa, approximately 14 million unintended pregnancies are recorded annually (3). Unintended pregnancy is common in low-income countries and has adverse pregnancy outcomes, with 42% of cases ending in miscarriage or abortion (4, 5). Unintended pregnancy is not only a problem for women with disabilities; it affects all of society, and thus, all pregnancies should be intended at the time of conception (6). In Ethiopia, the overall prevalence of unintended pregnancy among women of reproductive age is 30% (7, 8).

The World Health Organization (WHO) defines disability as a body impairment that makes it more difficult for a person to perform certain activities and interact with the world around them (9). However, according to the social model of disability, disability refers to the restriction caused by society when it does not give equivalent social and structural attention and support according to disabled people's structural needs (9, 10). The model is used to identify the systemic bottlenecks, degrading attitudes, and social exclusion that make it more difficult for women with disabilities to attain their daily functioning (11). It views disability as socially constructed marginalization, physical, social, programmatic, and attitudinal barriers experienced by women with disabilities. It argues that policies and practices should be tailored to women with disabilities (12–14).

The WHO global report on health equity reaffirms that health services should be provided based on free, informed consent and in an accessible and understandable manner to women with disabilities (15, 16). The United Nations Sustainable Development Goals emphasize achieving universal access to sexual and reproductive healthcare services and focus on improving equity to meet the needs of women and the most disadvantaged people (17). Despite these measures put in place, disability is a global public health problem because women with disabilities face numerous challenges in accessing sexual and reproductive services and experience more adverse pregnancy outcomes than those without disabilities (18–21).

Moreover, women with disabilities are predominantly disadvantaged in accessing healthcare services in the current facility-directed healthcare system (22). Studies have revealed that women with disabilities experience more significant unmet needs and inequities in accessing healthcare (23), and access to and experience of maternity care is suboptimal (24). Furthermore, inequities in access to sexual and reproductive health results in adverse pregnancy outcomes, thus posing a public health problem (25).

In the United States of America, a study revealed that more significant proportion (53%) of pregnancies are unintended among women with disabilities (26). In Ethiopia, studies revealed that the prevalence of unintended pregnancy among women with disabilities was approximately 67% (27) and 62.5% (28) in Addis Ababa and 65% in the Sidama National Regional State, respectively (29).

The literature has revealed that individual, interpersonal, community, and institutional level factors, like inaccessible facilities, healthcare providers’ insensitivity, a lack of knowledge about disabilities, physical violence, and reproductive coercion (30–33) were significant predictors for unintended pregnancy among women with disabilities.

In another study in Nepal, societal attitudes towards women with disabilities negatively affected the utilization of maternal healthcare services (34). In Uganda, the unfriendly physical structures and negative attitudes of service providers challenge women with disabilities to access SRH services (35). Studies in Ethiopia revealed that economic status, parity, residence, alcohol consumption, and knowledge of contraceptives were associated with unintended pregnancy (27, 29).

The prevalence and associated factors of unintended pregnancy among women without disabilities are well documented (7, 8, 36, 37), however, little is known about unintended pregnancy among women with disabilities in Ethiopia. Therefore, this study aimed to assess the prevalence of unintended pregnancy and associated factors among women with disabilities in the Central Regional State of Ethiopia. The study's findings provide insight into the prevalence and associated factors of unintended pregnancy among women with disabilities, and the researchers, programmers, and policymakers may use these findings as baseline data for interventions and strategies to improve accessibility of sexual and reproductive health services for all.

## Methods

### Study design, period, setting, and population

A community-based cross-sectional study was conducted from December 2023 to February 2024 in the Central Ethiopian regional state of Ethiopia. In the region, there are seven zones and three special districts. According to the National Statistical Agency population projection report (2023/24) the total population is 6,430,235; of these people, 3,243,411 (50.44%) are female, and 3,186,824 (49.56%) are male, and approximately 100,000 people are living with disabilities, of whom 50% are female. The region is the mostly rural, with 5,857,944 (91.1%) population living in rural and 572,291 (8.9%) population living in urban areas (38). There are 28 public hospitals, 22 health centers and 1,097 health posts. The target population consisted of all reproductive-age women with disabilities in the study area. Study population were selected pregnant women with disabilities from selected kebeles.

### Inclusion and exclusion criteria

Pregnant women with self-reported visual, hearing, speech, or physical disabilities who were permanent resident (live more than 6 months) in the selected cluster, as well as women who were pregnant at the time of data collection were eligible. Pregnant women with cognitive impairment, because they may have problems of remembering things and proving accurate information, as well as, women who were severely ill at the time of collection, were excluded from the study.

#### Sample size determination

A single population proportion formula was used to determine the sample size with the following assumptions: the proportion of unintended pregnancies from a previous study (*p* = 65.6%) (29), the reliability coefficient or critical value of the 95% confidence level (z = 1.96), and the degree of precision or margin of error (d = 5%)***^.^***Considering the design effect (d = 1.5) and nonresponse of 10%, the final sample included 572 participants.

#### Sampling procedure

The study employed multistage random sampling for participant enrollment. In the first step, three zones were selected randomly from the Central Regional State of Ethiopia. In the second stage, four districts were selected from each zone, twelve districts were included, and in the last stage, from each district, six kebeles were selected randomly, and seventy-two kebeles [clusters] were included in the final stage. The kebele is the smallest administrative unit of the Federal Democratic Republic of Ethiopia and a representative of the total population. The list of the women in the selected kebele's was compiled from the registration book kept by the health extension workers at the health post. A simple random sampling approach was used to choose the study participants. The sample size was proportionally allocated to each cluster based on the framework. The list of all pregnant women with disabilities in the selected clusters was obtained from registration book held by health extension workers in each cluster. Lastly, simple random sampling techniques were used to enroll study participants.

#### Study variables

The outcome variable was unintended pregnancy, and the predictor variables were individual, interpersonal, community, and institution-level factors.

### Outcome variable measurement

The London measurement of unplanned pregnancy conceptual framework has three dimensions: stance, context, and behaviour. Each dimension consists of two questions or items with 0, 1, or 2 scores. The scores were totalled over all six items, resulting in a score ranging from 0 to 12. However, in this study, pregnancy intention was assessed by asking the respondents about their intention and desire for a baby at conception. Those respondents who scored zero in the stance dimension, meaning they did not have a desire for pregnancy or had no expressed intention for pregnancy at the time of conception, were considered to have had an unintended pregnancy, as mentioned in Table 1 annexed (39, 40).

**Table 1:** The conceptual framework for unintended pregnancy among women with disabilities in the central regional state of Ethiopia (*N* = 562).

Dimensions	Questions	Score		
		0	1	2
Stance	Desire for pregnancy at time of conception	0	–	–
	Expressed intention for pregnancy at the conception	0	–	–
Context	Personal circumstances or timing	–	–	–
	Partner influences: agreement, desire for pregnancy	–	–	–
Behaviour	Pre-conceptual preparations (seeking health advice)	–	–	–
	Contraceptive use: (non-use, consistent use, methods failure)	–	–	–

### Data collection

A face to face interview was used to collect data using the Kobo Toolbox. The data collection tool was adopted, contextualized, and developed from previous studies and surveys (39, 41, 42). The tool consists of sociodemographic characteristics and individual, interpersonal, community, and institution-related variables. Women with physical or visual impairments were interviewed using structure questionnaires and got the response. However, women with hearing or speech impairments, along data collectors, sing or body language expertize were participated in translating the questions.

### Data analysis

Data were analyzed using the Statistical Package for Social Sciences (SPSS) version 26. The descriptive statistics are presented as frequencies and percentages in the tables and figures to show the distribution of predictor variables with the outcome variable. Bivariate logistic regression was used to select candidate variables and multiple logistic regression analyses were used to determine the associations of independent variables with the outcome variables. Multicollinearity was checked, and the Hosmer-Lemeshow test was used to check model fitness. Variables with *p* < 0.25 in the bivariate logistic regression were considered in the multivariable logistic regression model. The multivariable analysis used an adjusted odds ratio (AOR) with a 95% confidence interval (CI) and a *p*-value less than 0.05 to identify significant factors associated with unintended pregnancy.

### Ethical clearance and consent to participate

Ethical approval was obtained from the Research Ethical Committee of Wachemo University and the Research Advisory Committee of the Central University of Tamil Nadu, as referred to in Ref. No. WCU-IRB 0021/23. A permission letter was also obtained from the zonal and district health bureaus, and the Labour and Social Affairs offices. The participants were informed about the study's purpose and potential risks and benefits. Informed consent was obtained from each study participant, and confidentiality was ensured by keeping the data anonymous. By putting a signature or using their fingerprint on the consent form, participants have given their approval.

## Results

### Sociodemographic characteristics

In this study, 572 women with disabilities were enrolled for data collection. Five hundred sixty-two (562) participants responded to the questionnaire completely, resulting in a response rate of 98.2%. The mean age of the study participants was 30 (±3.6) years, and most respondents were between 25 and 34 years old. The participants’ ages ranged from 18 to 41 years. Nearly half (50%) of the study participants did not have formal education, and three hundred seventy-four (66.5%) had no occupation, as presented in Table 2.

**Table 2:** Sociodemographic characteristics of pregnant women with disabilities in the Central Regional State of Ethiopia 2024 (*N* = 562).

Variable	Categories	Frequency	Percentage
Age	<=24	61	10.9
	25–34	381	67.8
	>= 35	120	21.4
Marital status	Married	450	80.1
	Other^a^	112	19.9
Women's education level	No formal education	283	50.4
	Primary & Secondary	253	45.0
	Tertiary and above	26	4.6
Husband education level	No formal education	100	17.8
	Primary & Secondary	307	54.6
	Tertiary and above	43	7.7
Residence	Rural	459	81.7
	Urban	103	18.3
Religion	Protestant	374	66.5
	Orthodox	110	19.6
	Muslin	75	13.3
	Other^b^	3	0.5
Women occupation	No occupation	374	66.5
	Employed	45	8.0
	Private business	143	25.4
Husband's occupation	No occupation	141	25.1
	Employed	60	10.7
	Private business	249	44.3
Household wealth index	Low	462	82.2
	Medium	100	17.8
	High	0	0
Household size	≤5	386	68.7
	> 5	176	31.3

^a^ Single, divorced, widowed or separated.

^b^ Catholic, traditional religion.

### Individual and interpersonal level characteristics

Most study participants (66.9%) had never heard about pregnancy intentions, and three hundred seventy-two (66.2%) participants reported that male partners had a role in pregnancy intentions. Two hundred thirty (40.9%) of the respondents were prim gravida (first pregnancy), while two hundred ninety-two (87.9%) had low multiparty (less than or equal to five births). Among the study participants, 148 (44.6%) and 88 (26.5) had previously experienced unintended pregnancy and abortion, respectively (Table 3).

**Table 3:** Individual and interpersonal level characteristics of pregnant women with disabilities in the Central Regional State of Ethiopia (*N* = 562).

Variables	Categories	Frequency	Percentage
Ever heard about pregnancy intention	Yes	376	66.9
	No	186	33.1
Male partners have a role in pregnancy intention	Yes	372	66.2
	No	190	33.8
First pregnancy	Yes	230	40.9
	No	332	59.1
Parity^a^	1–4	292	87.9
	5–8	40	12.1
Ever experienced unintended pregnancy^a^	Yes	148	44.6
	No	184	55.4
Ever experienced abortion^a^	Yes	88	26.5
	No	244	73.5
History of stillbirth^a^	Yes	45	13.6
	No	287	86.4
History of contraceptive use	Yes	223	39.7
	No	339	60.3
Desire to have more children	Yes	138	24.6
	No	424	75.4

^a^ Indicates the denominator is women who had two or more pregnancy.

### Community and health facility level characteristics

Of the 562 respondents, 238 (40.6%) reported experiencing domestic violence, and 347 (61.7%) said that maternity services are inaccessible and unavailable to women with disabilities. Regarding transportation, 415 (73.8%) of the participants reported that the service was not accessible and inclusive. Furthermore, 391 (69.6%) responded that health facilities are inaccessible and insensitive to women with disabilities (Table 4).

**Table 4:** Community and health facility level characteristics of pregnant women with disabilities in the Central Regional State of Ethiopia (*N* = 562).

Variables	Categories	Frequency	Percentage
Ever experienced domestic violence	Yes	228	40.6
	No	334	59.4
Maternity service accessing or available	Yes	215	38.3
	No	347	61.7
Transport service accessible or inclusive	Yes	147	26.6
	No	415	73.8
Time spent to reach health facilities	≤ 1 h	65	15.7
	> 1 h	350	84.3
Disability-friendly health facilities structure	Yes	171	30.4
	No	391	69.6
The disparity in service delivery towards WWD	Yes	402	71.5
	No	160	28.5
Community negative attitude to WWD affects service uptake	Yes	450	80.1
	No	112	19.9

WWD, represents women with disabilities.

### Prevalence of unintended pregnancy among women with disabilities

**Figure 1:**
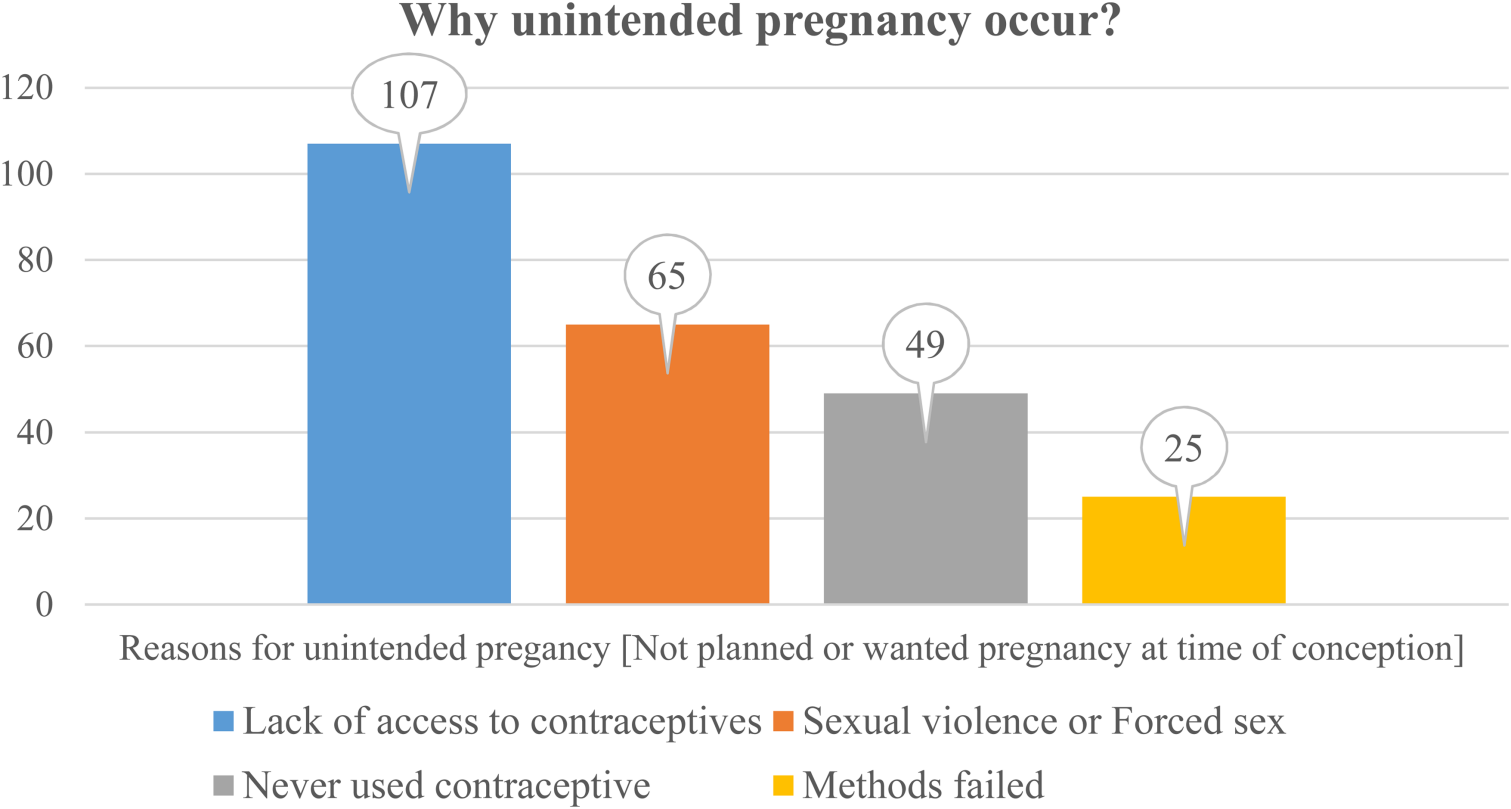
Reasons for unintended pregnancy among pregnant women with disabilities in the Central Regional State of Ethiopia (*n* = 246).

In this study, among the study participants, two hundred forty-six (43.8%) 95% CI: 39.5, 47.8 were unintended pregnancies at the time of conception, and three hundred sixteen (56%) were intended (planned or wanted) pregnancies. The self-reported reasons by the study participants for unintended pregnancy were lack of access to contraceptive services (43.5%), never using contraceptive methods (19.9%), sexual violence or forced sex (26.4%), and contraceptive failure (10.2%) (Figure 1).

### Factors associated with unintended pregnancy among women with disabilities

Multicollinearity of independent variables was checked, and the Hosmer_Lemeshow test was done to check the model's fitness. Variables with *p* values <0.25 on binary logistic regression were candidates for the final model. Multivariable logistic regression analysis revealed that unintended pregnancy was significantly associated with household size (AOR = 4.6, 95% CI: 2.6, 7.9, *p* < 0.0001), heard about pregnancy intention (AOR = 2.4, 95% CI:1.4, 4.1, *p* = 0.001), domestic violence (AOR = 5.9, 95% CI: 3.4, 10.4, *p* < 0.0001), accessibility of service (AOR = 2.4, 95% CI: 1.2, 5.4, *p* = 0.029), disability-friendly health facilities (AOR = 1.5, 95% CI: 1.2, 2.40, *p* = 0.040), discrimination by care providers (AOR = 2.1, 95% CI: 1.5, 2.9, *p* < 0.0001) and community attitudes (AOR = 2.7, 95% CI: 1.7, 4.3, *p* < 0.0001) among women with disabilities as presented in Table 5. The overall direction of the associated variables’ effect was positive.

**Table 5:** Factors significantly associated with unintended pregnancy among pregnant women with disabilities in the Central Regional State of Ethiopia (*N* = 562).

Variable	Unintended pregnancy		95%CI for Exp. (B)		*P*-value
	No (*N*,%)	Yes (*N*,%)	COR	AOR	
Marital status					
Married	304 (54.1)	146 (26.0)	0.60 (0.31, 0.91)	0.50 (0.12, 1.2)	
Others^a^	12 (2.1)	100 (17.8)	1.00	1.00	
Household size					
<=5	1,254 (45.2)	32 (23.5)	1.00	1.00	
>5	62 (11.0)	114 (20.3)	3.5 (2.4, 5.1)	4.6 (2.6, 7.9)**	0.000
Heard about pregnancy intentions					
Yes	264 (47.0)	112 (112)	1.00	1.00	
No	52 (9.30)	134 (23.8)	6.07 (4.1, 8.96)*	2.4 (1.4, 4.1)**	0.001
First pregnancy					
Yes	140 (24.9)	90 (16.0)	1.00	1.00	
No	176 (31.3)	156 (27.8)	1.4 (0.98, 1.94)*	1.3 (0.7, 2.3)	
Ever experienced domestic violence					
Yes	46 (8.2)	182 (32.4)	16.7 (10.3, 20.4)*	5.9 (3.4, 10.4)**	0.000
No	270 (48.0)	64 (11.4)	1.00	1.00	
Maternity service accessibility					
Yes	155 (27.6)	60 (10.7)	1.00	1.00	
No	161 (28.6)	186 (33.1)	2.9 (2.1, 4.3)	2.4 (1.2, 5.4)**	0.029
Disability-friendly health facilities structure					
Yes	115 (20.5)	56 (10.0)	1.00	1.00	
No	201 (35.8)	190 (33.8)	1.9 (1.3, 2.8)*	1.5 (1.2, 2.40)	0.040
Disability inclusive transportation					
Yes	101 (18.0)	46 (8.2%)	2.0 (1.4, 3.04)*	0.70 (0.42, 1.20)	
No	215 (38.3)	200 (35.6)	1.00		
Discrimination by care providers towards WWD					
Yes	138 (24.6)	157 (27.9)	2.3 (1.6, 3.2)	2.1 (1.5, 2.9)**	0.000
No	178 (31.7)	89 (15.8)	1.00	1.00	
Community attitude towards WWD					
Yes	280 (49.8)	170 (30.2)	1.00	1.00	
No	36 (6.40)	76 (13.5)	3.5 (2.2, 5.4)*	2.7 (1.7, 4.3)	0.000

^a^ Single, widowed, divorced.

* *P* < 0.25.

** *P* < 0.05 (indicates statistical significance).

## Discussion

From the perspective of the social model of disabilities, this study showed that factors at the individual, interpersonal, community and institutional levels are associated with unintended pregnancy among women with disabilities. The prevalence of unintended pregnancy among women with disabilities was 43.8% (95% CI: 39.5, 47.8%). This finding was higher than that of a study conducted in Ghana (6.4%) (43). However, these values are lower than those reported in studies conducted in Addis Ababa [62.5% (28) and 67% (27), respectively], in the Sidama Regional State 65.6% (29) Ethiopia, and in the United States of America, 53% (26). A possible explanation for the discrepancy in the findings might be variations in the sociodemographic characteristics (e.g., education, occupation, economic status, and residence) of the respondents, study time, and differences in approach and outcome variable measurement.

In terms of household size, women with more than five household members are approximately five times more likely to become pregnant unintentionally than women with fewer household members. This finding is similar to prior research in Gambia (44); nevertheless, a study in Ethiopia found that women with five or fewer children are more likely to have unintended pregnancies (45). The inconsistent findings may be attributed to differences in the study area and the demographics (e.g., age, marital status, education, employment, and income) of the study participants.

Unintended pregnancy is significantly predicted by women's awareness of their aim to become pregnant. Compared to women who have heard about pregnancy intentions, those who have never heard of pregnancy intentions are two times more likely to become pregnant unintentionally. Other studies in Gondar (46) and Addis Ababa, Ethiopia (27), support this conclusion. One possible explanation for this could be that respondents who have ever heard of pregnancy intentions may be more aware of ways to prevent unintended pregnancies. Another explanation is that women who are unaware of pregnancy intentions could become pregnant unwittingly.

Domestic violence is a public health issue, and this study revealed that it was significantly associated with unintended pregnancy. Women who had experienced sexual violence and reproductive coercion were nearly six times more likely to become pregnant inadvertently as compared to their counterparts. This conclusion is consistent with previous findings that sexual violence is a significant predictor of unplanned pregnancy (27, 47, 48). One possible explanation is that sexual assault increases the likelihood of unintended pregnancy. In certain circumstances, women find it difficult to report that they have been assaulted. During this period, women may be vulnerable to unintended pregnancy and other complications.

Women with disabilities encounter several difficulties accessing sexual and reproductive health services, including transportation, improper examination settings, a lack of ramps, and inaccessible restrooms in health facilities (35, 49, 50). In this study, the accessibility of services was found to be a significant predictor of unintended pregnancy. Unintended pregnancy was twofold more likely to occur in respondents who said sexual and reproductive services were inaccessible as compared to their counterparts. This finding is supported by previously conducted studies that revealed that inaccessibility doubled the burden risk of unintended pregnancy (32, 51–54). One possible explanation is that denying women with disabilities access to sexual and reproductive health services can have several detrimental effects, such as unplanned pregnancies and their complications. This result leads to the conclusion that accessing sexual and reproductive health services is the best strategy for ensuring universal health coverage.

Many women with disabilities are discouraged from seeking health care since the majority of health facilities are not disability-inclusive. In this study, disability-unfriendly health facilities were significantly associated factors for unintended pregnancy. Those women who reported that health facilities were disability-unfriendly were nearly twice as likely to become pregnant inadvertently. Other studies have shown similar results (49, 55, 56). A possible reason might be that women may not use sexual and reproductive health services at nearby health facilities unless they are disability-inclusive. As a result, women may have limited access to or awareness of birth control techniques, leading them to become pregnant unknowingly.

Discrimination and exclusion are everyday experiences for women with disabilities, preventing them from receiving timely, high-quality care. This study revealed that discrimination in healthcare access was strongly associated with unintended pregnancy among women with disabilities. Care provider attitudes influence service uptake, and respondents who claimed injustice in healthcare services were twice as likely to have an unintended pregnancy. This conclusion is corroborated by earlier research findings (57–59). A possible reason might be that women with disabilities are less likely to receive sexual and reproductive health services from health facilities due to disparities in service delivery. Discriminatory services at health institutions prevent women from having equitable access to services that potentially increase the likelihood of unintended pregnancy.

Community attitudes can have a significant impact on the lives of people with disabilities. Furthermore, women's pregnancy intentions can be influenced by community stigma and ignorance about equal opportunities to access healthcare. In this study, negative community attitudes towards reproductive matters among women with disabilities contributed threefold more to becoming pregnant unintendedly. This finding is supported by previous studies conducted in different countries (47, 60, 61). A possible explanation might be that community negative attitudes have a significant impact on the use of sexual and reproductive health services by women with disabilities. Therefore, unless all stockholders work hard to change community attitudes, women with disabilities cannot benefit equally from the service. As a result, this approach might contribute to reducing the risk of unplanned pregnancy and related complications among women with disabilities.

### Implication of the study

According to the study's findings, extra commitment and attention from all stockholders to sexual and reproductive health services is required to meet the special needs of women with disabilities and achieve universal sexual and reproductive health coverage under the SDG agenda. More research is required to explore the depth perceived factors and experience of unintended pregnancy from the perspectives of healthcare providers and women with disabilities.

### Strengths and limitations of the study

The study findings could be input for policymakers and national/international organizations in reducing the burden of unintended pregnancies among women with disabilities. Since the study used a cross-sectional design, it couldn't establish a causal relationship between outcome variables and predictors as well as explore the depth of participants’ experiences and perspectives. There might be recall biases and social desirability biases because data collectors were health extension workers. Women with cognitive disabilities were excluded, which may affect the generalizability of the findings.

## Conclusion

This study sheds light on the prevalence and associated factors of unintended pregnancy, as well as the need for disability-inclusive healthcare services. Therefore, to achieve universal access to sexual and reproductive healthcare targeted under the Sustainable Development Goals, the Minister of Women and Social Affairs, Ministry of Health, Regional Health Bureau, and health facilities authorities should pay attention to improving the awareness on pregnancy intention and domestic violence and ensuring accessible, inclusive, and equitable maternal health care for women with disabilities. Further longitudinal studies are recommended to investigate the causality of predictors and outcome variable, as well as qualitative studies to explore the depth of lived experience of unintended pregnancy among women with disabilities.

## Data Availability

The dataset presented in this study can be found in the article or Supplementary material, further inquiries can be directed to the corresponding author.
